# Impact of Bispectral Index- and Target-Controlled Infusion-Guided Sedation on Patient Comfort and Procedural Workflow During Bronchoscopy

**DOI:** 10.3390/healthcare13172218

**Published:** 2025-09-04

**Authors:** Ya-Chi Chang, Kun-Ta Lee, Ching-Yi Chen, I-Jung Liu, Yun-Kai Yeh, Kuan-Yuan Chen, Po-Hao Feng, Cheng-Yu Tsai

**Affiliations:** 1 Respiratory Therapy Room, Division of Pulmonary Medicine, Taipei Medical University-Shuang Ho Hospital, New Taipei City 23561, Taiwan; 09480@s.tmu.edu.tw (Y.-C.C.); 09481@s.tmu.edu.tw (K.-T.L.);; 2 Research Center of Sleep Medicine, College of Medicine, Taipei Medical University, Taipei 11031, Taiwan; a05459@tmu.edu.tw; 3 Division of Pulmonary Medicine, Department of Internal Medicine, Taipei Medical University-Shuang Ho Hospital, New Taipei City 23561, Taiwan; 4 Division of Pulmonary Medicine, Department of Internal Medicine, School of Medicine, College of Medicine, Taipei Medical University, Taipei 11031, Taiwan; 5 TMU Research Center for Thoracic Medicine, Taipei Medical University, Taipei 11031, Taiwan; 6 Graduate Institute of Clinical Medicine, College of Medicine, Taipei Medical University, Taipei 11031, Taiwan; 7 School of Respiratory Therapy, College of Medicine, Taipei Medical University, Taipei 11031, Taiwan; 8 Sleep Center, Taipei Medical University-Shuang Ho Hospital, New Taipei City 23561, Taiwan; 9 TMU Research Center of Artificial Intelligence in Medicine, Taipei Medical University, Taipei 11031, Taiwan; 10 School of Biomedical Engineering, College of Biomedical Engineering, Taipei Medical University, Taipei 11031, Taiwan

**Keywords:** bronchoscopy, Target-Controlled Infusion, Bispectral Index, sedation, procedural workflow

## Abstract

**Objectives**: While a combination of the Target-Controlled Infusion (TCI) system and Bispectral Index (BIS) monitoring has emerged as an effective approach for sedation management during bronchoscopy, its benefits and potential risks remain insufficiently explored. In this study, we evaluated the impacts of the BIS- and TCI-guided sedation systems on patient comfort and procedural workflow during bronchoscopy. **Methods**: A prospective observational study was conducted at a single tertiary medical center. Patients scheduled for diagnostic bronchoscopy were assigned to either a sedation group or a control group, and the sedation group received BIS- and TCI-guided sedation using propofol and fentanyl. Objective time metrics such as the procedure time, observation time, and overall total time were recorded. Subjective outcomes including pain, discomfort, and fear levels were assessed using a visual analogue scale (range: 0–10). Operator-rated procedural difficulty was also assessed. **Results**: The use of a BIS- and TCI-guided sedation system did not significantly affect the procedure duration but led to prolonged observation and overall total times. Patients in the sedation group reported significantly lower pain, coughing, and breathlessness sensations and overall discomfort levels compared to the control group. Both pre- and post-procedural fear levels were lower in the sedation group. Operator-rated procedural difficulty was significantly lower in the sedation group, and operator-reported difficulty was significantly correlated with both endoscopic insertion and coughing sensations. **Conclusions**: These findings support that the BIS- and TCI-guided sedation system may improve patient experiences and optimize procedural workflow during bronchoscopy. Future randomized studies with larger and more-diverse populations are recommended to enhance the robustness and generalizability of the results.

## 1. Introduction

Bronchoscopy is widely used as a diagnostic and therapeutic tool in pulmonary medicine, particularly for evaluating airway lesions [1], performing biopsies, and staging lung cancer [2,3]. Despite its clinical value, conventional bronchoscopy performed under local anesthesia can induce considerable patient discomfort, including coughing, pain, and anxiety [4,5]. These reactions not only affect the individual experience but also may interfere with the procedural quality [6]. Such side effects can disrupt visualization, prolong examination times, and compromise the accuracy of tissue acquisition [7].

To address these limitations, conscious sedation techniques have increasingly been adopted. Among them, the use of the Target-Controlled Infusion (TCI) system in combination with Bispectral Index (BIS) monitoring allows for precise adjustment of the sedation depth during bronchoscopy [8,9]. Technically, TCI delivers agents such as propofol and fentanyl based on individualized pharmacokinetic modeling, while BIS provides real-time neurophysiological feedback. By maintaining BIS values within the suggested moderate sedation range, adequate sedation can be achieved without progressing to deep anesthesia, thereby minimizing patient movement and optimizing procedural efficiency [10,11]. This sedation protocol is designed to enhance both patient comfort and procedural efficiency.

Nevertheless, concerns remain regarding potential side effects and safety considerations associated with sedation during bronchoscopy, particularly when agents such as propofol or fentanyl are used. One of the most commonly cited risks is prolonged recovery times [12], which can delay discharge and impact procedural throughput, especially in high-volume centers. In addition, patients with underlying comorbidities such as fatty liver and obesity were independently linked to an increased risk of excessive sedation and delayed recovery [13,14]. Researchers have also reported transient oxygen desaturation and the need for airway interventions during conscious sedation bronchoscopy [15], especially in elderly patients or those with sleep apnea or pre-existing pulmonary disease [16,17].

Beyond physiological risks, psychological and emotional factors may also influence decisions about undergoing sedation. Some individuals express hesitation or refusal due to anxiety or prior negative experiences with sedative drugs [18]. Others may worry about potential decreased cognition, post-sedation grogginess and pain, or a sense of losing control during the procedure [19]. Cultural beliefs and mistrust in sedative medications, along with inadequate knowledge about anesthesia, can further contribute to reluctance [20]. These emotional concerns might not only affect patient cooperation and satisfaction [21], but also influence dosage requirements and increase the risks of adverse effects [22]. This underscores the importance of thorough pre-procedure counseling and shared decision-making [23]. Thus, objective data—particularly on time-related procedural metrics, sedation depth consistency, and patient-reported outcomes such as fear, pain, and comfort—may shed light on the comprehensive impacts of sedation during bronchoscopy. However, such investigations remain limited, and further research is needed to elucidate the benefits and risks of evidence-based sedation practices.

In this study, we compared procedural characteristics—including operator-rated difficulty and time-related metrics—between sedated and non-sedated bronchoscopy. In addition, patient-reported outcomes were assessed using structured questionnaires to capture subjective experiences such as pain, fear, and comfort. By integrating both objective clinical parameters and subjective patient feedback in this study, we sought to comprehensively evaluate the impact of the BIS- and TCI-guided sedation system on bronchoscopy tolerability, procedural difficulty, and overall clinical performance.

## 2. Materials and Methods

### 2.1. Ethical Approval

This study was conducted following ethical approval granted by the Institutional Review Board of Taipei Medical University (TMU-Joint Institutional Review Board approval no. TMU-N202305091 and approval date: 29 June 2023). All research procedures were implemented in accordance with ethical standards outlined in the *Declaration of Helsinki*. This includes the collection, anonymization, analysis, and storage of patient data, all of which were carried out in full compliance with the approved research protocol. As the research posed minimal risk and did not adversely affect the rights or welfare of participants, the requirement for informed consent was waived by the IRB.

### 2.2. Research Flow and Participant Enrollment

**Figure 1:**
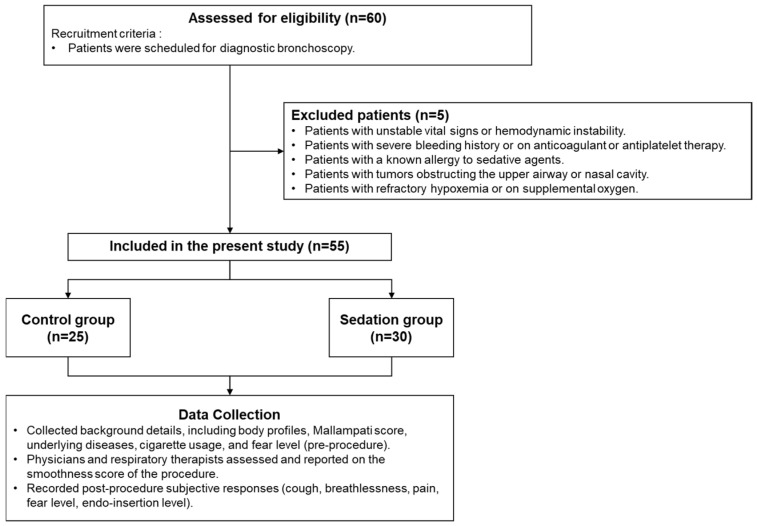
Research flow for recruiting participants.

This prospective observational study was conducted at Taipei Medical University–Shuang Ho Hospital, a tertiary medical center located in northern Taiwan. Adult patients scheduled for diagnostic bronchoscopy (i.e., autofluorescence bronchoscopy or bronchoalveolar lavage) were recruited between February and October 2023. The research flow for recruiting participants is presented in Figure 1. Exclusion criteria included those with (1) unstable vital signs or hemodynamic instability; (2) a history of severe bleeding or current use of anticoagulant or antiplatelet therapy; (3) a known allergy to sedative agents; (4) the presence of tumors obstructing the upper airway or nasal cavity; and (5) with refractory hypoxemia or current oxygen support. All eligible participants were thoroughly informed about the objectives and procedures of the present study. Subsequent medical assessments were conducted, and relevant clinical history data were retrieved from medical records.

Figure 1 outlines participant enrollment and data collection processes for our study. Initially, 60 hemodynamically stable patients from the Department of Chest Medicine, not on supplemental oxygen, were assessed. Five patients were excluded due to conditions such as unstable vital signs, allergy to sedative agents, refractory hypoxemia, severe bleeding risk, or tumors obstructing the upper airway. The remaining 55 were divided into two groups: 25 in the control group and 30 in the sedation group. Data collection included demographics, Mallampati score, underlying diseases, cigarette usage, and pre-procedure body fat levels. Procedural difficulty was assessed by physicians and respiratory therapists, while post-procedure responses―including coughing, pain, and endoscopic insertion sensations, as well as breathlessness, fear, and discomfort levels―were recorded.

### 2.3. Group Classification and Data Collection

Performing physicians initially assessed the Mallampati score for enrolled patients to evaluate the upper airway visibility, using the standard classification based on the structures while the patient was in a supine position [24]. To respect patient autonomy and standard clinical practice, participants elected to undergo bronchoscopy either with sedation (self-funded) or without sedation, based on clinical recommendations and their individual preferences. Each patient made this decision independently, without influence from this research.

Regarding the subjective outcome evaluation, questionnaires incorporating a visual analogue scale (VAS; range: 0–10) were used. Specifically, all questionnaires were self-administered by patients to minimize recall bias, with standardized instructions provided by assessors to ensure consistent understanding without influencing responses. Participants were asked to rate their anticipated level of fear prior to the procedure, while attending physicians and nursing staff assessed coughing severity during bronchoscopy. After the procedure, participants again completed VAS assessments, assessing the post-procedure fear level, coughing sensation, discomfort level, pain sensation, endoscopic insertion sensation, and the breathlessness level. Meanwhile, the performing physicians independently assess their perception of procedural difficulty (scale: 0–10, from smooth to difficult). Additionally, objective time-related metrics—including procedure time, post-anesthesia recovery time, observation time, and overall total time—were recorded for further analysis. Definitions of these time metrics are presented in Table 1.

### 2.4. Sedation Procedure and Dosage Maintenance

During bronchoscopy, sedation depth was continuously monitored using the BIS system (PHILIPS IntelliVue MX500, Amsterdam, The Netherlands). The target BIS value was maintained at 60–70, corresponding to an appropriate level of moderate sedation [25,26]. Peripheral oxygen saturation was simultaneously monitored, with a target value above 90% to ensure adequate respiratory function throughout the procedure. If the BIS value exceeded the desired range—indicating insufficient sedation—the TCI system (Fresenius Kabi Injectomat^®^ TIVA Agilia, Midrand, South Africa) automatically adjusted the infusion rate by delivering additional doses of propofol or fentanyl as needed. This closed-loop adjustment enabled precise control of the sedation depth, minimized patient movement and discomfort, and allowed for smoother execution of the procedure using a flexible bronchoscope (Olympus BF-Q290, Tokyo, Japan) [27]. All dosing modifications were guided by real-time BIS feedback and performed under the supervision of trained medical personnel to prevent over-sedation and its associated risks.

### 2.5. Statistical Examination

All statistical analyses were performed using Python (vers. 3.9.23, Python Software Foundation, Fredericksburg, VA, USA) along with the open-source library Scikit-learn (vers. 1.6.1). The Shapiro–Wilk test was applied to assess the normality of the distribution of continuous variables. For normally distributed variables, comparisons between the groups with and without sedation were conducted using Student’s *t*-test, while the Mann–Whitney U-test was employed for variables that did not meet normality assumptions. Differences in categorical variables were evaluated using either the Chi-squared test or Fisher’s exact test, depending on expected cell counts. To examine associations between VAS scores and operator-rated procedural smoothness, Spearman’s rank correlation analysis was utilized. Multiple linear regression analyses were then performed, adjusting for potential cofounding factors, including sex and lung cancer subtype. Effect sizes were estimated using Cohen’s d, and post-hoc power analyses were conducted at a significance level of α = 0.05. A two-tailed *p* value of <0.05 was considered statistically significant.

## 3. Results

### 3.1. Baseline Demographics and Clinical Features

Table 2 presents the demographic and clinical characteristics of both groups. In total, 55 patients were enrolled in this study and classified into a control group (*n* = 25) and a sedation group (*n* = 30) based on their decision to undergo bronchoscopy with or without procedural sedation. The mean age of participants in the control group was 59.72 ± 14.23 years, compared to 66.67 ± 11.51 years in the sedation group, with no statistically significant difference (*p* = 0.11). Similarly, there was no significant difference in the sex distribution between groups (*p* = 0.07), although a higher proportion of males was observed in the control group (80.0%; 20 males, 5 females) compared to the sedation group (53.3%; 16 males, 14 females).

Regarding body profiles and upper airway evaluation, no significant differences were observed between the groups in terms of the body-mass index (BMI), neck circumference, or Mallampati score. For lung disease distribution, pneumonia was the most prevalent condition in both the control group (72%) and sedation group (36.67%), while 50% of patients in the sedation were diagnosed with lung cancer. Similarly, cigarette usage patterns were comparable between the groups (*p* = 0.44), with the majority of participants being never-smokers (control group: 64%; sedation group: 46.67%).

### 3.2. Procedure-Related Time Metrics and Procedural Difficulty

Mean doses of propofol and fentanyl administered in the sedation group were 153.56 ± 72.06 mg and 0.11 ± 0.01 mg, respectively (Table S1). Table 3 summarizes comparisons of procedure-related time metrics and operator-reported procedural difficulty from attending physicians between the control and sedation groups. The mean procedure time was slightly longer in the sedation group (27.47 ± 11.33 min) than in the control group (22.20 ± 9.58 min), although the difference did not reach statistical significance (*p* = 0.06). Post-anesthesia recovery time, as expected, was applicable only to the sedation group, with a mean duration of 11.13 ± 9.65 min. The observation time was significantly longer in the sedation group (12.47 ± 5.62 min) compared to the control group (7.21 ± 6.78 min, *p* < 0.01). Consequently, the overall total time spent in the procedural unit was also significantly greater in the sedation group (49.73 ± 15.57 min) than in the control group (31.8 ± 13.3 min, *p* < 0.01).

A similar pattern was observed in the subgroup analysis of patients without lung cancer (Table S2). Both the observation time (11.73 ± 5.80 vs. 6.88 ± 6.73 min) and overall total time (48.47 ± 17.66 vs. 30.83 ± 12.65 min) were significantly longer in the sedation group (*n* = 15) compared to the control group (*n* = 24; both *p* < 0.01).

In terms of procedural difficulty assessed by the VAS, operators reported significantly smoother experiences in the sedation group, as indicated by lower difficulty scores (1.43 ± 1.72) compared to the control group (2.60 ± 2.42, *p* < 0.05), with higher scores reflecting greater procedural difficulty. However, among patients without lung cancer, the difference was not statistically significant (1.47 ± 1.73 vs. 2.67 ± 2.44, Table S2).

### 3.3. Subjective Responses to the Procedure

Table 4 summarizes the subjective questionnaire responses collected from participants in both the control and sedation groups. Pain sensation scores were significantly lower in the sedation group (0.03 ± 0.18) compared to the control group (2.24 ± 2.45, *p* < 0.01). Similar trends were observed in breathlessness level (0.03 ± 0.18 vs. 1.60 ± 2.65, *p* < 0.01), endoscopic insertion sensation (0.17 ± 0.59 vs. 4.60 ± 2.96, *p* < 0.01), and coughing sensation (0.77 ± 1.19 vs. 5.96 ± 3.09, *p* < 0.01). In contrast, the control group reported significantly higher discomfort levels (5.44 ± 2.96) compared to the sedation group (0.40 ± 0.89, *p* < 0.01), indicating that sedation was associated with greater patient comfort. Regarding fear levels, the sedation group reported significantly reduced fear both before (1.87 ± 2.37 vs. 3.76 ± 2.42, *p* < 0.01) and after (0.60 ± 1.57 vs. 3.88 ± 3.02, *p* < 0.01) the procedure compared to the control group. Effect size estimations are presented in Table S3, and all outcomes exhibited large effect sizes with high achieved power.

Similar trends were observed among patients without lung cancer (*n* = 39, Table S4). Compared to the sedation group, the control group reported significantly higher pain sensations (0.0 ± 0.0 vs. 2.25 ± 2.51), endoscopic insertion sensations (0.27 ± 0.80 vs. 4.79 ± 2.86), and coughing sensations (1.00 ± 1.51 vs. 6.21 ± 2.89, all *p* < 0.01). Overall discomfort was also greater in the control group (5.58 ± 2.93 vs. 0.47 ± 1.13, *p* < 0.01). Fear levels were consistently higher both before (3.83 ± 2.44 vs. 2.20 ± 2.86, *p* < 0.05) and after the procedure (3.96 ± 3.06 vs. 1.00 ± 2.14, *p* < 0.01).

### 3.4. Correlations Between Operator-Rated Difficulty and Patient-Reported Experiences

**Figure 2:**
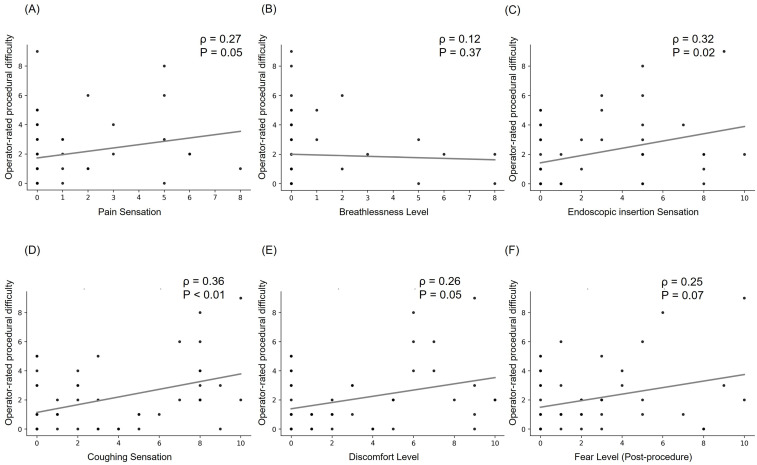
Correlations between operator-rated difficulty and patient-reported sensations. Each panel presents a specific sensation: (**A**) pain sensation, (**B**) breathlessness level, (**C**) endoscopic insertion sensation, (**D**) coughing sensation, (**E**) discomfort level, and (**F**) fear level (post-procedure). Spearman correlation coefficients (ρ) and corresponding significance levels (*p*) are displayed to indicate the strength and statistical significance of each correlation.

Results derived from the Spearman correlation analysis between operator-rated procedural difficulty and various subjective patient responses are illustrated in Figure 2. Significant positive correlations were observed between the perceived procedural difficulty and several indicators of patient discomfort. Specifically, higher difficulty ratings were moderately correlated with a greater endoscopic insertion sensation (ρ = 0.32, *p* < 0.05) and coughing sensation (ρ = 0.36, *p* < 0.01). In terms of emotional responses, higher difficulty ratings tended to be associated with elevated post-procedure fear levels (ρ = 0.25) and discomfort levels (ρ = 0.26), although these correlations did not reach statistical significance. Similarly, pain sensation and breathlessness levels showed non-significant trends toward being correlated with procedural difficulty.

### 3.5. Associations Between Sedation Use and Subjective Responses

Table S5 presents associations between sedation use and subjective responses reported by patients and operators, adjusting for sex and lung cancer subtype. Sedation was significantly associated with reduced patient-reported ratings for pain sensation (−1.97, 95% CI: −3.06 to −0.89, *p* < 0.01), endoscopic insertion sensation (−4.33, 95% CI: −5.67 to −3.00, *p* < 0.01), breathlessness level (−1.50, 95%CI: −2.70 to −0.30, *p* < 0.05), and coughing sensation (−4.80, 95% CI: −6.28 to −3.32, *p* < 0.01). Regarding emotional responses, sedation was also significantly associated with a lower discomfort level (−4.97, 95% CI: −6.36 to −3.59) and post-procedure fear level (−3.17, 95% CI: −4.69 to −1.66, both *p* < 0.01). Conversely, there was no significant association between sedation and the operator-rated procedural difficulty.

## 4. Discussion

The BIS- and TCI-guided sedation system has potential as a precise tool for adjusting the sedation depth during bronchoscopy. However, its effects on procedural efficiency, patient-reported experiences, and overall clinical utility remain insufficiently explored. In this study, we collected and compared both objective metrics and subjective outcomes from patients who underwent bronchoscopy with or without sedation. The procedure-related time metrics were significantly longer in the sedation group compared to the control group, whereas operator-rated procedural difficulty was lower in the sedation group. Patients who received BIS- and TCI-guided sedation also reported more-favorable experiences than those in the control group, characterized by lower levels of pain, fear, and discomfort. Operator-rated procedural difficulty was significantly correlated with patient-reported endoscopic insertion and coughing sensations. Similar results were observed in the multiple regression models, adjusted for sex and lung cancer subtype.

Age, BMI, neck circumference, and Mallampati score were comparable between the sedation and control groups. However, the control group included a higher proportion of male participants, which may be attributed to sex-related differences in pain tolerance. Women are known to exhibit greater pain sensitivity and report pain more frequently than men, particularly in cases of muscle-skeletal or neuropathic pain [28,29]. In the present study, group assignment was based on clinical recommendations and individual preferences, which potentially contributed to an unequal distribution of sex between the groups. Regarding procedure-related time metrics, the use of sedation did not significantly affect the duration of the bronchoscopy itself but lengthened the observation and overall total times. As aforementioned, extended recovery and monitoring are well-documented side effects associated with sedation [30]. In the present study, the extended times may be primarily attributable to additional monitoring protocols to ensure patient safety, and no adverse events were observed with sedation use. Conversely, operators reported lower procedural difficulty in the sedation group, highlighting the potential clinical utility of the BIS- and TCI-guided sedation system in facilitating smoother bronchoscopy. This finding aligns with a prior study that indicated reduced procedural interference—such as patient movement or coughing—when a BIS-guided propofol infusion was performed [31]. Additionally, TCI-guided sedation provides precise and steady delivery of sedative agents, allowing individualized dose adjustments in real time [32]. Taken together, applying the BIS- and TCI-guided sedation system during bronchoscopy shows promise in enhancing procedural control and optimizing overall efficiency.

Patients in the sedation group reported significantly less pain and coughing sensations along with lower levels of discomfort and fear compared to those in the control group. Although fear of uncertainty and losing control may impede the decision to undergo sedation, the sedation group generally reported a more-positive procedural experience. The interplay between psychological and physical factors may partially account for these findings. Previous research demonstrated that preoperative anxiety of patients is correlated with both the required dose of sedative agents and the length of the postoperative recovery [33]. Additionally, pre-bronchoscopy anxiety was identified as a predictor of procedural discomfort, independent of the total procedure duration [34]. In our study, although the correlation between operator-rated procedural difficulty and the patient-reported discomfort level did not reach statistical significance, a positive trend was observed. Moreover, the multivariate regression model indicated that improvements in patient experiences and comfort levels with sedation were independent of cofounding factors. Collectively, these results suggest that sedation during bronchoscopy may reduce patient discomfort and optimize the procedural workflow, especially when guided by the BIS and TCI system. From a cost-effectiveness perspective, although sedation is typically self-funded, its potential to facilitate the procedure, enhance patient experience, and reduce discomfort and fear may outweigh the additional expense.

This study has some strengths. First, it provides a comprehensive evaluation of the BIS- and TCI-guided sedation system in bronchoscopy by incorporating both objective time metrics and subjective responses related to procedural experiences and patient comfort. Next, reduced operator-rated difficulty was correlated with fewer sensations of coughing and endoscopic insertion during bronchoscopy reported by patients. In addition, the use of sedation was associated with lower patient-reported sensations and discomfort. These findings highlight the clinical utility of sedation in optimizing procedural control, and potentially help inform the development of evidence-based strategies to improve clinical practice. Furthermore, the results support that integration of a BIS- and TCI-guided sedation system may be a feasible approach to enhance patient experiences and reduce procedural complexity. However, the relatively small and homogeneous sample from a single medical center may have limited the generalizability of these findings. Additionally, the non-randomized, non-blinded design and patient self-selection of sedation may have introduced natural selection bias, influenced subjective responses, and potentially led to placebo or nocebo effects, thereby affecting the clinical utility of the results. Although both objective and subjective parameters were included to provide a comprehensive perspective, the potential contributions of counseling and psychological support in alleviating patient anxiety and fear were not evaluated. Future research with larger, more-diverse populations, employing randomized and blinded design and incorporating non-pharmacological strategies, is warranted to enhance the methodological rigor and strengthen the external validity of BIS- and TCI-guided sedation systems.

## 5. Conclusions

To provide a comprehensive evaluation of the BIS- and TCI-guided sedation system in a clinical bronchoscopy setting, this study integrated both objective metrics and self-reported scores related to procedural experience and efficiency. The findings suggest that the use of a BIS- and TCI-guided sedation system in bronchoscopy may enhance patient experience while simultaneously optimizing the procedural workflow. Future randomized studies with larger, more-diverse populations, incorporating evaluation of psychological support strategies, are warranted to strengthen the generalizability and further validate these findings.

## Figures and Tables

**Table 1:** Definitions of the time metrics.

Time Metric	Definition
Procedure time	The interval from the start of bronchoscope insertion to the completion of bronchoscopy.
Post-anesthesia recovery time	The period from the end of sedation administration to the point at which the patient regained full consciousness, could respond clearly to verbal questions, and demonstrated stable vital signs.
Observation time	The time from awakening until the patient was considered clinically ready to leave the recovery area, marked by cessation of coughing, stable vital signs, independent ambulation, and the ability to follow instructions.
Overall total time	The total duration from the initiation of the procedure to the time the patient exited the procedural or recovery area.

**Table 2:** Comparisons of demographic characteristics between the control and sedation groups.

Variable	Control Group (*N* = 25)	Sedation Group (*N* = 30)	*p*
Age (years) ^b^	59.72 ± 14.23	66.67 ± 11.51	0.11
Sex (male/female) ^c^	20/5	16/14	0.07
BMI (kg/m^2^) ^a^	22.94 ± 3.84	22.35 ± 3.85	0.57
Neck circumference (cm) ^a^	37.51 ± 3.27	35.55 ± 4.38	0.07
Mallampati score (level: 1–4) ^b^	2.48 ± 0.71	2.31 ± 0.92	0.29
Lung disease (*n*, %)			
Pneumonia	18 (72%)	11 (36.67%)	
Asthma	1 (4%)	-	
Bronchitis	1 (4%)	1 (3.33%)	
Interstitial lung disease	-	1 (3.33%)	
COPD	2 (8%)	1 (3.33%)	
Tuberculosis	2 (8%)	1 (3.33%)	
Lung cancer (*n*, %)			
Adenocarcinoma	1 (4%)	9 (30%)	
Squamous cell carcinoma	-	4 (13.33%)	
Pulmonary sarcoma	-	1 (3.33%)	
Small cell carcinoma	-	1 (3.33%)	
Cigarette usage (*n*, %) ^c^			0.44
Never	16 (64%)	14 (46.67%)	
Previously quit	3 (12%)	5 (16.67%)	
Current smoker	6 (24%)	11 (36.66%)	

Abbreviations: BMI, body-mass index; COPD, chronic obstructive pulmonary disease. Data are expressed as the mean ± standard deviation or number (%). ^a^ Differences between groups were assessed using Student’s *t*-test. ^b^ Differences between groups were assessed using the Mann–Whitney U-test. ^c^ Differences between groups were assessed using the Chi-squared test.

**Table 3:** Comparisons of procedure-related time metrics and operator-reported procedural difficulty between the control and sedation groups.

Variable	Control Group (*N* = 25)	Sedation Group (*N* = 30)	*p*
Procedure-related time parameters (min)			
Procedure time	22.20 ± 9.58	27.47 ± 11.33	0.06
Post-anesthesia recovery time	-	11.13 ± 9.65	-
Observation time	7.21 ± 6.78	12.47 ± 5.62	<0.01
Overall total time	31.8 ± 13.3	49.73 ± 15.57	<0.01
Subjective response (VAS score: 0–10)			
Operator-rated procedural difficulty	2.60 ± 2.42	1.43 ± 1.72	<0.05

Abbreviations: VAS, visual analogue scale. Data are expressed as mean ± standard deviation. Differences between groups were assessed using the Mann–Whitney U-test.

**Table 4:** Comparison of patient-reported subjective sensations between the control and sedation groups.

Variable (VAS Score: 0–10)	Control Group (*N* = 25)	Sedation Group (*N* = 30)	*p*
Pain sensation	2.24 ± 2.45	0.03 ± 0.18	<0.01
Breathlessness level	1.60 ± 2.65	0.03 ± 0.18	<0.01
Endoscopic insertion sensation	4.60 ± 2.96	0.17 ± 0.59	<0.01
Coughing sensation	5.96 ± 3.09	0.77 ± 1.19	<0.01
Discomfort level	5.44 ± 2.96	0.40 ± 0.89	<0.01
Fear level (pre-procedure)	3.76 ± 2.42	1.87 ± 2.37	<0.01
Fear level (post-procedure)	3.88 ± 3.02	0.60 ± 1.57	<0.01

Abbreviations: VAS, visual analogue scale. Data are expressed as the mean ± standard deviation. Differences between groups were assessed using the Mann–Whitney U-test.

## Data Availability

All data were collected between February and October 2023 at Taipei Medical University–Shuang Ho Hospital. The dataset is not available as Supplementary Materials, due to the inclusion of personal information. Requests for access to the data or related documents can be directed to the corresponding author.

## Supplementary Materials

The following supporting information can be downloaded at https://www.mdpi.com/article/10.3390/healthcare13172218/s1; Table S1: Propofol and Fentanyl Dosages in the Sedation Group (N = 30). Table S2: Comparison of Procedure-Related Time Metrics and Operator-Rated Procedural Difficulty Among Patients Without Lung Cancer (N = 39). Table S3: Effect Size Calculation and Statistical Power Analysis of Patient-Reported Subjective Sensations between the Control and Sedation Groups. Table S4: Comparison of Patient-Reported Subjective Sensations Among Patients Without Lung Cancer (N = 39). Table S5: Associations Between Sedation Use and Subjective Responses.

## Abbreviations

The following abbreviations are used in this manuscript:

BIS	Bispectral Index
BMI	body-mass index
TCI	Target-Controlled Infusion
VAS	visual analogue scale
