# Supplementary material for: Impact of Bispectral Index- and Target-Controlled Infusion-Guided Sedation on Patient Comfort and Procedural Workflow During Bronchoscopy
Source: Healthcare (Basel). 2025 Sep 4;13(17):2218. doi: 10.3390/healthcare13172218 (PMC12427744; doi:10.3390/healthcare13172218)
Supplement: Supplementary file 1 [file healthcare-13-02218-s001.zip › healthcare-3801148-supplementary.pdf]

Supplementary Materials

Table S1. Propofol and Fentanyl Dosages in the Sedation Group (*N*=30).

Sedative agent	Mean ± SD (mg)	Range (mg)
Propofol	153.56 ± 72.06	49.3–333.0
Fentanyl	0.11 ± 0.01	0.05–0.63

Abbreviations: SD, standard deviation.

Table S2. Comaprison of Procedure-Related Time Metrics and Operator-Rated Procedural Difficulty Among Patients Without Lung Cancer (*N*=39)

**Variable**	**Control group (*N*=24)**	**Sedation group (*N*=15)**	***p***
**Procedure-related time parameters** (min)			
Procedure time	21.46 ± 9.03	27.40 ± 13.08	0.18
Post-anesthesia recovery time	-	12.00 ± 10.72	-
Observation time	6.88 ± 6.73	11.73 ± 5.80	<0.01
Overall total time	30.83 ± 12.65	48.47 ± 17.66	<0.01
**Subjective Response** (VAS score: 0–10)			
Operator-rated procedural difficulty	2.67 ± 2.44	1.47 ± 1.73	0.12

Abbreviations: VAS, visual analogue scale.
Data are expressed as the mean ± standard deviation.
Differences between groups were assessed using the Mann-Whitney U-test.

Table S3. Effect Size Calculation and Statistical Power Analysis of Patient-Reported Subjective Sensations between the Control and Sedation Groups

Variable (VAS score: 0–10)	Control group (*N*=25)	Sedation group (*N*=30)	Cohen's d	Power
Pain sensation	2.24 ± 2.45	0.03 ± 0.18	1.17	> 0.99
Breathlessness level	1.60 ± 2.65	0.03 ± 0.18	0.79	0.95
Endoscopic insertion sensation	4.60 ± 2.96	0.17 ± 0.59	1.89	> 0.99
Coughing sensation	5.96 ± 3.09	0.77 ± 1.19	2.04	> 0.99
Discomfort level	5.44 ± 2.96	0.40 ± 0.89	2.00	> 0.99
Fear level (pre-procedure)	3.76 ± 2.42	1.87 ± 2.37	0.78	0.94
Fear level (post-procedure)	3.88 ± 3.02	0.60 ± 1.57	1.23	> 0.99

Abbreviations: VAS, visual analogue scale.

Data are expressed as the mean ± standard deviation.

Table S4. Comaprison of Patient-Reported Subjective Sensations Among Patients Without Lung Cancer (*N*=39)

Variable (VAS score: 0–10)	Control group (*N*=24)	Sedation group (*N*=15)	*p*
Pain sensation	2.25 ± 2.51	0.0 ± 0.0	<0.01
Breathlessness level	1.67 ± 2.68	0.0 ± 0.0	<0.01
Endoscopic insertion sensation	4.79 ± 2.86	0.27 ± 0.80	<0.01
Coughing sensation	6.21 ± 2.89	1.00 ± 1.51	<0.01
Discomfort level	5.58 ± 2.93	0.47 ± 1.13	<0.01
Fear level (pre-procedure)	3.83 ± 2.44	2.20 ± 2.86	<0.05
Fear level (post-procedure)	3.96 ± 3.06	1.00 ± 2.14	<0.01

Abbreviations: VAS, visual analogue scale.
Data are expressed as the mean ± standard deviation.
Differences between groups were assessed using the Mann-Whitney U-test.

Table S5. Associations Between Sedation Use and Subjective Responses

**Variable (VAS score: 0–10)**	Beta coefficient (95% confidence interval)	***p***
Pain sensation	-1.97 (-3.06 to -0.89)	<0.01
Breathlessness level	-1.50 (-2.70 to -0.30)	<0.05
Endoscopic insertion sensation	-4.33 (-5.67 to -3.00)	<0.01
Coughing sensation	-4.80 (-6.28 to -3.32)	<0.01
Discomfort level	-4.97 (-6.36 to -3.59)	<0.01
Fear level (post-procedure)	-3.17 (-4.69 to -1.66)	<0.01
Operator-rated procedural difficulty	-0.94 (-2.31 to 0.43)	0.17

Abbreviations: VAS, visual analogue scale.

Multivariable linear regression models were adjusted for sex and lung cancer subtype.
